# α,β-Unsaturated (Bis)Enones as Valuable Precursors in Innovative Methodologies for the Preparation of Cyclic Molecules by Intramolecular Single-Electron Transfer

**DOI:** 10.3390/molecules31030430

**Published:** 2026-01-26

**Authors:** Tommaso Benettin, Francesca Franco, Fabrizio Medici, Sergio Rossi, Alessandra Puglisi

**Affiliations:** Dipartimento di Chimica, Università degli Studi di Milano, via Golgi, 19, 20133 Milan, Italy; tommaso.benettin@unimi.it (T.B.); francesca.franco@unimi.it (F.F.); fabrizio.medici@unimi.it (F.M.); sergio.rossi@unimi.it (S.R.)

**Keywords:** α,β-unsaturated (bis)enones, cyclic molecules, radicals, single-electron transfer (SET), cyclization reactions, photochemical reactions, photocatalytic reactions, metal-driven reactions, electrochemical reactions

## Abstract

The synthesis of monocyclic and bicyclic compounds plays a fundamental role in organic chemistry, and the need for novel synthetic methodologies is still under investigation. In particular, α,β-unsaturated (bis)enones have emerged as valuable precursors for the formation of cyclic (both mono and bicyclic) structures through single-electron transfer (SET) processes. Single-electron transfer (SET) is a redox process where one electron moves from a donor species to an acceptor, generating radical ions or neutral radicals that drive unique reaction pathways. Thanks to the advent of radical chemistry, it was possible to discover an entirely new reactivity of α,β-unsaturated (bis)enones, which, after a SET event, undergo the formation of cyclic molecules, both in intra and inter-molecular reactions, under several possible pathways, including formal [2+2] cycloaddition reaction (22CA) and 5-exo-trig cyclization, for ring closure. Today, the generation of radical species can be broadly classified into three main approaches: *photochemical* and *photocatalytic*, *metal-driven* and *electrochemical* processes. In this review, we summarize the progress achieved to date in the synthesis of cyclic molecules from α,β-unsaturated (bis)enones via single-electron transfer events under these three main classes of processes. Whenever possible, the reaction pathway and fate of the radical species generated through SET is discussed.

## 1. Introduction

**Figure 1:**
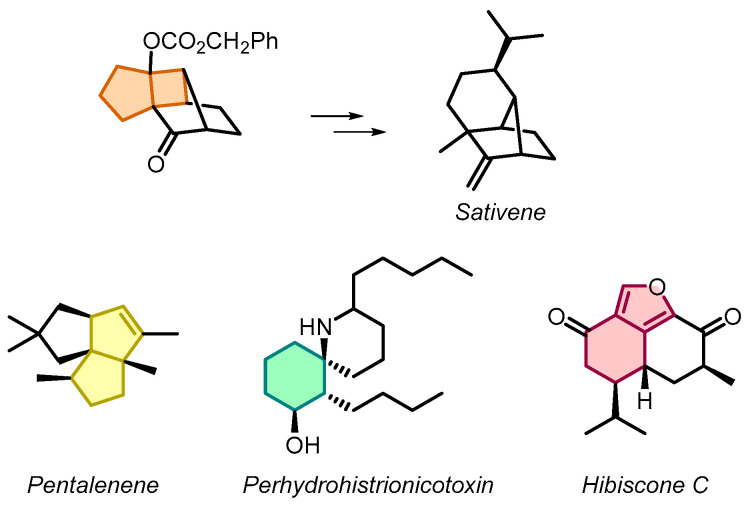
Examples of ring-opening reactions from bicyclo[3.2.0]heptanes to generate natural products and cyclic natural products.

In organic chemistry, the synthesis of monocyclic and bicyclic compounds is funda-mental, both for proving the reactivity of compounds and for the design of complex functional molecules. From a molecular synthesis perspective, monocyclic rings often serve as convenient building blocks and synthetic intermediates. At the same time, bicyclic systems offer access to three-dimensional molecular shapes, enhanced rigidity and defined stereochemistry [1]. In particular, bicyclic compounds can be used as key intermediates for the synthesis of different bioactive molecules or natural products. An example of these features is the bicyclo[3.2.0]heptanes that are used as versatile compounds for ring-opening, rearrangement and functionalization processes [2]. One example is reported in Figure 1: Sativene, a compound belonging to an important family of fungal sesquiterpenoids, can be obtained by a ring-opening reaction and consequent manipulation of the corresponding bicyclo[3.2.0]heptane. Moreover, the prevalence of mono- and bicyclic frameworks in biologically active compounds and many natural products such as Pentalenene [3], Perhydrohistrionicotoxin [4] and Hibiscone C [5] underscores their practical importance (Figure 1).

For these reasons, the discovery of novel synthetic methodologies for the cyclization of molecules in organic chemistry remains a challenge. In this context, the employment of α,β-unsaturated (bis)enones has been particularly studied over the past two decades. Thanks to the advent of radical chemistry, it was possible to discover an entirely new reactivity of these compounds, which, after a single-electron transfer (SET), undergo the formation of cyclic molecules, both in intra and inter-molecular reactions. Single-electron transfer (SET) is a redox process where one electron moves from a donor species to an acceptor, generating radical ions or neutral radicals that drive unique reaction pathways This mechanism plays a fundamental role not only in organic chemistry synthesis [6] but also in biosynthesis [7], chemical biology [8] and material science [9].

**Scheme 1:**
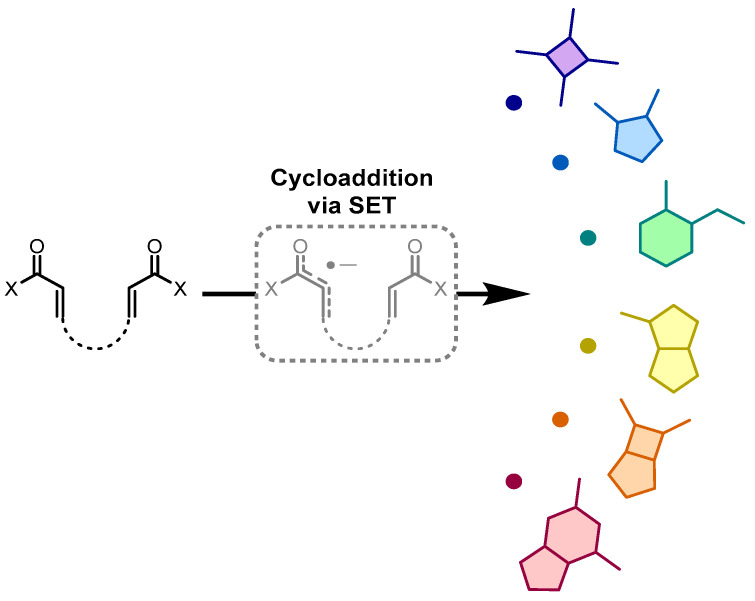
Direct access to different types of cyclo-membered rings from α,β-unsaturated ketones via single electron transfer (SET).

This strategy, compared to a direct excitation of the double bond, led to achieving four, five or six-membered rings, or even different types of bicyclic molecules, easily and under mild conditions (Scheme 1).

**Figure 2:**
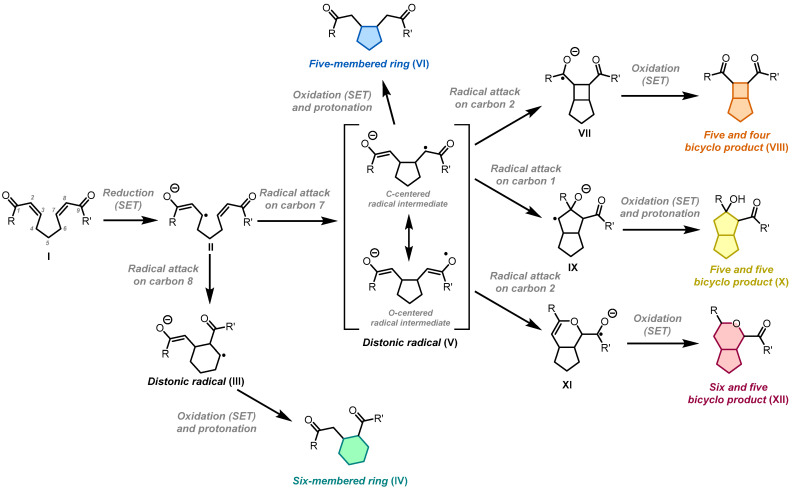
Possible cyclization pathways of α,β-unsaturated ketones after SET reduction.

Since the discovery of the triphenylmethyl radical (considered the first organic free radical) in 1987 by Gomberg [10] to today, the understanding of reactivity, behavior and stability of molecules containing an open-shell atom in organic chemistry has led to enormous interest by synthetic chemists. Several processes have been discovered and designed, but the formation of radical species today can be classified into three main classes: *photochemistry* and *photocatalytic* processes, *metal-driven* processes and *electrochemical* processes [11]. In this review, the state of the art in the preparation of cyclic molecules by single electron transfer using α,β-unsaturated (bis)enones as starting compounds under photocatalytic, electrochemical and metal-driven conditions will be presented. The methodologies for the cyclization driven by the direct excitation or energy transfer mechanisms of these molecules will not be discussed. For these topics, these reviews [12,13] are recommended. As previously mentioned, radical chemistry offers several possible pathways for ring closure in α,β-unsaturated ketone systems. These various cyclization processes will be described and discussed in the following sections. However, for a clearer understanding, all the possible mechanisms leading to the formation of the different ring systems reviewed in this work are summarized in Figure 2.

Radical generation occurs through an initial reduction step via SET to the α,β-unsaturated ketone substrate (**I**), leading to the formation of the radical anion intermediate (**II**). This intermediate can undergo two distinct cyclization pathways, depending on which carbon of the double bond participates in the radical attack. Specifically, if the radical attacks carbon 8 of the molecule, a six-membered ring is then formed (compound **IV**). On the other hand, if the attack occurs on carbon 7, a five-membered ring distonic radical is formed (intermediate **V**). From the five-membered distonic radical **V**, different pathways are possible, based on the radical delocalization. When the unpaired electron is localized on the carbon atom, the following three pathways can be accomplished:

(*i*) Oxidation of **V**, followed by protonation, affords the corresponding 5-exo-trig, five-membered ring product (**VI**).

(*ii*) The radical attack on carbon atom 2 leads to the formation of intermediate **VII**. Oxidation of **VII** through SET allows to obtain product **VIII**, a formal [2+2] cycloaddition reaction (22CA) product.

(*iii*) The radical attack on carbon atom 1 allows for the formation of intermediate **IX**, which then evolves to product **X**.

On the other hand, when the unpaired electron is localized on the oxygen atom of intermediate **V**, the radical attack on carbon atom 2 leads to intermediate **XI**, followed by oxidation to product **XII**.

In the following sections, we discuss the different methodologies (*photochemistry* and *photocatalytic*, *metal-driven* and *electrochemical*) reported in the literature in the last three decades in this field.

## 2. Photocatalytic Methodologies

Photocatalysis emerged as a powerful tool in organic chemistry, offering new pathways for the activation and transformation of organic molecules under mild and generally more sustainable conditions. Using photons as traceless reactants, it is possible to have access to high reactive species such as radicals or excited states. Photocatalytic systems can promote a wide range of reactions, including oxidations, reductions and C–C or C–N bond formations, that are often difficult to achieve through traditional thermal methods [14]. This light-driven approach has significantly expanded the synthetic toolbox of organic chemists, enabling more efficient and selective synthesis of complex molecules while reducing environmental impact. In this context, photocatalysis played a key role in developing novel methodologies for the reduction of α,β-unsaturated carbonyl compounds [13,15,16].

**Scheme 2:**
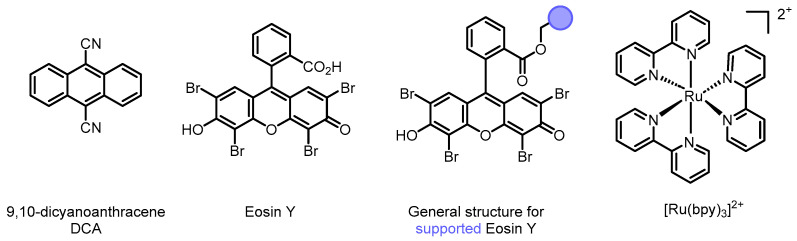
Photocatalyst structures.

To help the reader, we have reported the structures of the photocatalysts mentioned in the paper (Scheme 2).

**Scheme 3:**
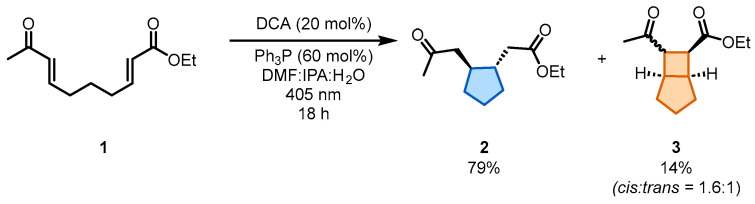
Reductive cyclization of compound **1** promoted by 9,10-dicyanoanthracene (DCA) as photocatalyst.

The first example of the photocatalyzed cycloaddition of bis α,β-unsaturated carbonyl compound **1** (Scheme 3) was reported by Pandey et al. in 1997 [17]. This study aimed to develop a novel methodology to exploit visible light to perform electron transfer processes and, in this way, to harvest photons into electrons and utilize them for triggering one-electron reductive β-activation of α,β-unsaturated ketones for radical reactions, changing their reactivity from their usual tendency to accept radical attacks to a more nucleophilic character. The pioneering work in this context demonstrated that, by carefully selecting photocatalysts based on their redox potentials, it was possible to activate the α,β-unsaturated enones by single-electron transfer (SET). To prove this concept, the authors described a photosystem using 9,10-dicyanoanthracene (DCA) as the so-called visible-light-harvesting electron acceptor and triphenyl phosphine as the sacrificial electron donor (ED). After 18 h of irradiation, a complete conversion of the starting α,β-unsaturated ketone **1** (Scheme 3) was obtained, a 79% yield of the *trans*-5-exo-trig product **2** was obtained and a 14% yield of a 1.6:1 mixture of the **3**-*cis*:**3**-*trans* bicyclic byproducts was obtained (*cis*:*trans*, 1.6:1).

**Scheme 4:**
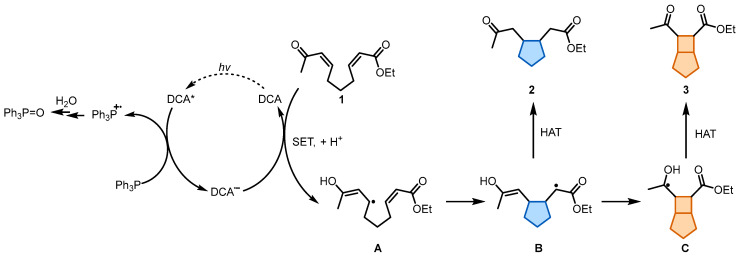
Proposed mechanism for the reductive cyclization of compound **1**.

The proposed mechanism (shown in Scheme 4) reports that under light irradiation (405 nm), the DCA is promoted to its excited state DCA* and can undergo a single electron transfer with the Ph_3_P, forming the DCA radical anion (DCA^·−^). This species is then able to reduce the α,β-unsaturated ketone **1** through a single electron transfer (SET), regenerating the neutral form of DCA and achieving the desired radical centered at the β-position of the starting ketone moiety. The so-obtained radical intermediate **A** undergoes an intramolecular radical attack to give the radical intermediate **B**. At this point, the radical intermediate can yield the monocyclic product **2** by H-abstraction or undergo a second intramolecular radical attack, losing a hydrogen atom and yielding the bicyclic product **3**.

The authors also studied the factors governing the regio and stereoselectivity of the products of this reaction. The major product obtained is the 5 exo-trig compound **2**, as *trans* isomer only. The ratio of the monocyclic product to bicyclic product has to be ascribed to the higher stability of intermediate **B** compared to **C**, favoring the HAT process over further cyclization.

**Scheme 5:**
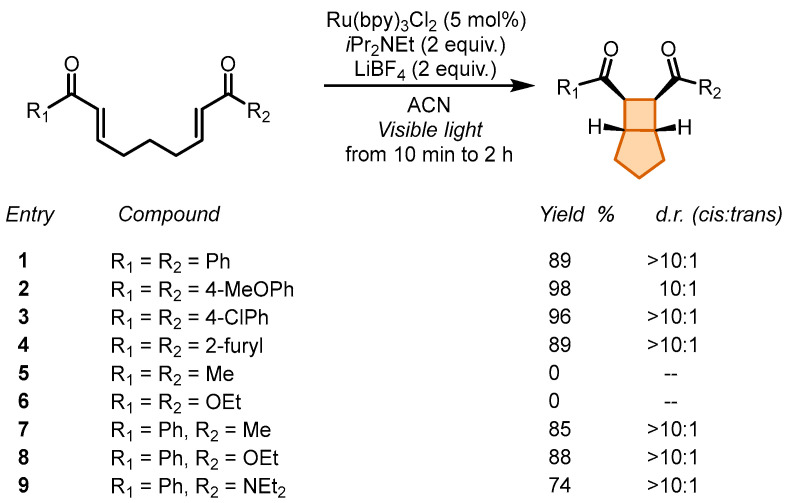
The [2+2] photocycloaddition of α,β-unsaturated ketones promoted by Ru-based photocatalyst.

In 2008, Yoon and his group published a paper in which the use of ruthenium complexes to promote photo-driven transformation to achieve the [2+2] cycloaddition of different types of (bis)enones substrates (Scheme 5) was studied [18]. The reaction was performed with 5 mol% of Ru(bipy)_3_Cl_2_ as a photocatalyst, 2 equivalents of *i*-Pr_2_NEt and 2 equivalents of LiBF_4_ as an additive in acetonitrile. They achieved a good to excellent yield (from 54% to 98%) of the bicyclic adduct by irradiating the solution with a 275 W floodlight.

Interestingly, the reaction worked under sunlight irradiation through a laboratory window as the sole source of irradiation. Exploring the scope of this reaction, it was found that symmetrical aryl (bis)enones bearing electron-donating and electron-withdrawing substituents are both excellent substrates for cycloaddition, as are heteroaryl enones (entries 1–4, Scheme 5). On the other hand, aliphatic (bis)enones and enoates do not cyclize (entries **5** and **6**): a result that the authors attribute to the more negative reduction potential of the aliphatic symmetric bisenones. This hypothesis was further supported by the cycloadditions of unsymmetrical bisenones, in which the presence of one aryl moiety appears to be a strict requirement. The mechanism of the reaction was also studied with different control experiments, proving the need of *i*-Pr_2_NEt for the reductive quenching of the photoexcited state (Ru(bipy)_3_^2+^*) to form a Ru(bipy)_3_^+^ complex that can transfer an electron to the lithium-activated bisenone.

**Scheme 6:**
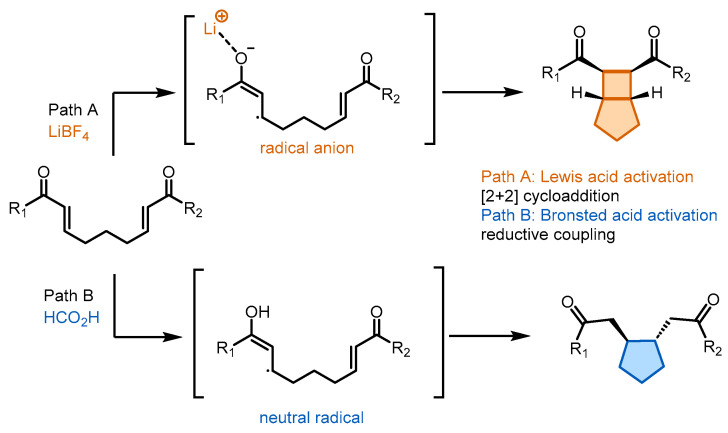
Effect of Lewis and Brønsted acids in the photocatalytic reductive cyclization of enones, using ruthenium complex.

Starting from the work of Pandey [17] and the unexpected formation of both the 5-*exo*-trig cyclization product **2** (Scheme 3) and the bicyclic compound **3** (Scheme 3) as products, Yoon’s group studied how the effects of the presence or the absence of a negative charge in an unpaired spin in the enone radical anion can drive the regioselectivity of the photocatalyzed cyclization reaction [19]. The group found that the presence of both a charge and an unpaired spin in the enone radical anion is mandatory for the formation of the cyclobutane adduct, whereas the 5-*exo*-trig cyclization reactivity would be achieved with the neutral form of the radical intermediate. After figuring out this mechanistic aspect, the group developed different protocols to selectively achieve the two products (Scheme 6, Path **A** and **B**).

**Scheme 7:**
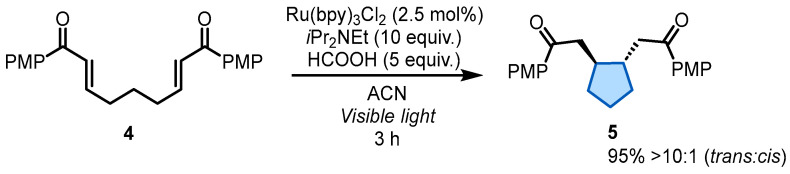
Reductive coupling cyclization of compound **4**.

In particular, activation of bisenone with a Brønsted acid, HCO_2_H, instead of a Lewis acid, LiBF_4_, would afford a cationic oxocarbenium species, which, after reduction, evolved into the neutral radical intermediate (Scheme 6, Path **B**). After fine-tuning the conditions for the synthesis of the monocyclic products, starting from compound **4** (Scheme 7) as the model substrate, it was found that using 2.5 mol% Ru(bpy)_3_Cl_2_, 5 equivalents HCO_2_H and 10 equivalents *i*-Pr_2_NEt allowed to achieve the 5-*exo*-trig product **5** in 95% yield after 3 h of irradiation.

Interestingly, as in Pandey’s work, the cyclization only resulted in the formation of the *trans* stereoisomer. Moreover, the authors studied the differences between the two activation protocols. First, the scope of each reaction is quite distinct; while neither aliphatic enones nor styrenes participate in the radical anion [2+2] cycloaddition, they are excellent reaction partners in the radical-mediated reductive cyclization process. Second, the two cyclization events favor divergent stereochemical outcomes; while the [2+2] cycloaddition requires a *cis* ring junction, the reductive cyclization of enones is generally *trans*-selective.

**Scheme 8:**
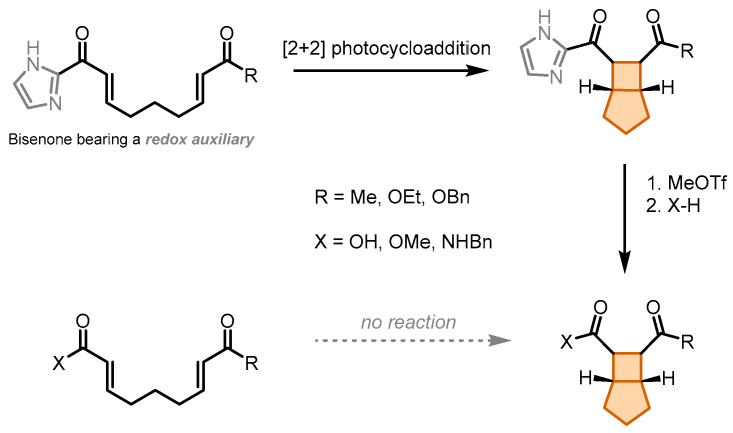
The [2+2] photocycloaddition of bisenones bearing the redox auxiliary.

In order to overcome the limits of the applicability of the [2+2] cycloaddition protocols, Yoon’s group installed a cleavable auxiliary group, called redox auxiliary [20], onto the (bis)enone substrate to facilitate the one-electron reduction and subsequent cycloaddition of the substrate. Notably, the redox auxiliary could be transformed into a carboxylic acid, ester, amide or similar carbonyl-containing functional group by late-stage functionalization, as highlighted in Scheme 8.

**Scheme 9:**
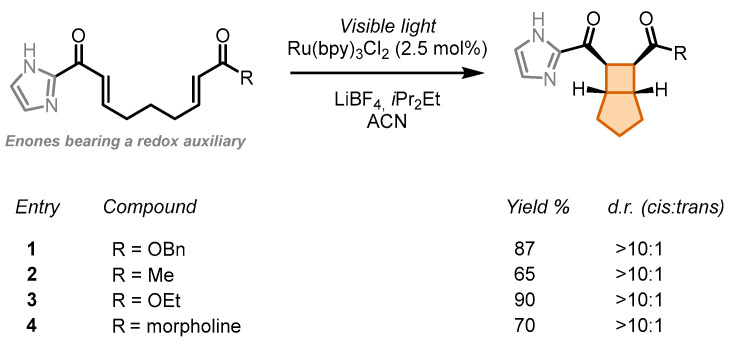
Scope for the intramolecular [2+2] photocycloaddition of bisenones bearing the redox auxiliary.

Using heteroaryl groups facilitates the reduction of the (bis)enone substrate to the key radical anion intermediate required for cycloaddition, which is then susceptible to cleavage with a variety of nucleophiles under mild conditions. This protocol works well for both intramolecular and intermolecular cyclobutanation reactions, achieving good to excellent results in terms of yields and diastereoselectivity (up to >10:1 *cis*:*trans*). The reaction tolerates a variety of functional groups, such as esters, aliphatic ketones and amides (Scheme 9).

**Scheme 10:**
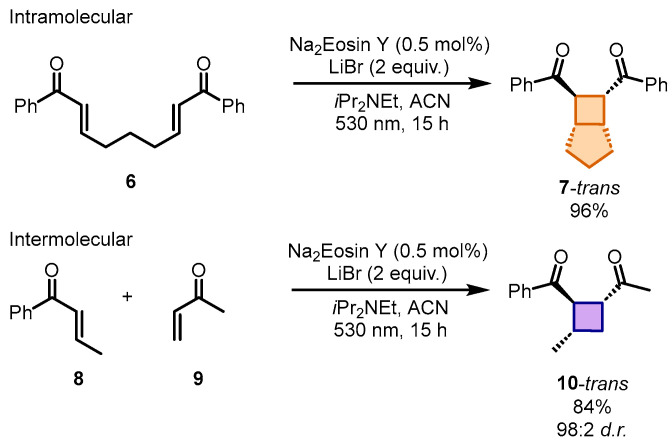
Intra- and intermolecular cyclization of aryl (bis)enones promoted by Na_2_Eosin Y as photocatalyst.

Taking inspiration from the work of Yoon on how different Bronsted or Lewis acids lead to different cyclization products after SET reduction of (bis)enones, Zeitler’s group also contributed to this field by exploring the possibility of replacing metal photoredox complexes with organic dyes (Scheme 10) [21]. Her research group used Eosin Y sodium salt as an alternative for [Ru(bpy)_3_]Cl_2_. The group first demonstrated that Eosin Y sodium salt can act as a photocatalyst to promote the [2+2] cycloaddition of aryl enones for both intra- and intermolecular reactions, achieving excellent yields by irradiating the reaction mixture for 15–24 h with a 530 nm LED. Interestingly, contrary to Yoon’s research, they obtained the complete opposite diastereoselectivity of the cyclobutanation reaction and the *trans* stereoisomer was selectively obtained, with a *d.e.* of up to 96%.

**Scheme 11:**
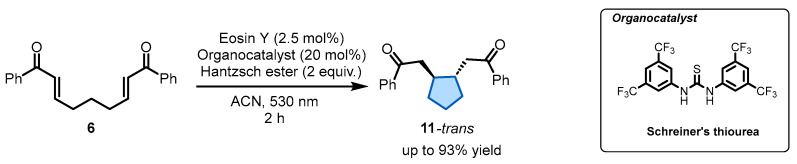
Reductive coupling of **6**, using Eosin Y as photocatalyst and Schreiner’s thiourea as H-bond donor.

Moreover, the group also focused on combining photoredox catalysis with H-bond donor catalysis in order to mimic the Bronsted acid activation of α,β-unsaturated enones. Using this type of activation, they were able to achieve excellent results in terms of yield (up to 95%) by using Schreiner’s thiourea and replacing the sacrificial reducing agent *i*-Pr_2_NEt with a Hantzsch ester, as illustrated in Scheme 11. Also in this case they achieved the *trans* 5-exo-trig cyclization product **11** selectively.

**Scheme 12:**
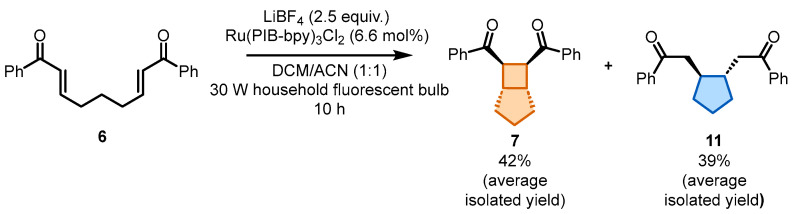
The [2+2] photocycloaddition of **6**, promoted by a polymer-supported Ru(II) photocatalyst.

To improve both efficiency and catalyst recovery, Bergbreiter’s group reported the first example of cycloaddition of aryl bis(enones) using a supported photocatalyst [22]. In particular, they investigated the possibility to use a polymer-immobilized photocatalyst as a recyclable heterogeneous system for a range of photochemical transformations, including the [2+2] cycloaddition of bisenones (Scheme 12). The authors reported that by employing a polyisobutylene (PIB)-ligated version of the Ru(II) bipyridine dichloride complex (Ru(PIB-bpy)_3_Cl_2_), it is possible to achieve the cycloaddition of the symmetric aryl bisenone **6**, achieving, a mixture of the two cyclized products after 10 h of irradiation: **7** and **11**. The catalyst can be reused up to 5 times without loss of reactivity, achieving compound **7** in a 42% average yield and compound **11** in a 39% yield.

**Scheme 13:**
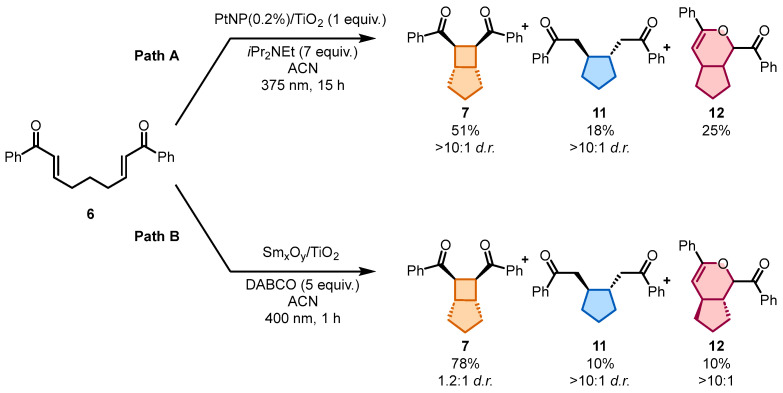
(Path **A**) [2+2] cycloaddition of **6**, using a Pt-Ti based heterogeneous photocatalyst (PtNP/TiO_2_). (Path **B**) [2+2] cycloaddition of **6**, using a Sm-Ti based dual photoredox Lewis acid catalyst (Sm_x_O_y_/TiO_2_).

To achieve the advantages of heterogeneous catalysis, like the high efficiency stemming from phase separation and catalyst reusability, Scaiano’s group demonstrated that the [2+2] photocycloaddition of (bis)enones could be conducted by using a heterogeneous photocatalyst and platinum nanoparticles supported on titania (PtNP/TiO_2_) as a photocatalyst (Scheme 13, Path **A**) [23]. Later on, the same authors developed one of the first examples of heterogeneous dual photoredox-Lewis acid catalysis, using a versatile and efficient nanocomposite: Sm_2_O_3_ nanoparticle-decorated titanium dioxide [24]. With this new heterogeneous catalyst, they were able to obtain the desired bicyclic product **7** in a higher selectivity, with respect to the monocyclic product **11**, compared to their previous work, due to the dual role of samarium oxide as a photoredox catalyst and Lewis acid (Scheme 13, Path **B**).

However, if the use of a heterogeneous dual catalyst favored the formation of the bicyclic product **7** (Scheme 13, Path **B**), a lower diastereoselective ratio *cis*:*trans* was achieved, probably due to a different coordination of the ketone moiety. The authors proposed that the heterogeneous catalytic mechanism for the major product closely follows that of the homogeneous catalytic system. The ketone moiety is initially coordinated to the Lewis acid samarium oxide, then a SET from the photoexcited nano composite catalyst to the activated substrate forms the key radical anion intermediate, stabilized by the Sm_x_O_y_ NPs. Subsequent intramolecular Michael addition leads to five-membered ring formation, followed by cyclobutanation to afford the samarium-coordinated ketyl radical. The authors suggest that the ketyl radical is then oxidized via SET to quench H^+^ in the photocatalyst.

**Scheme 14:**
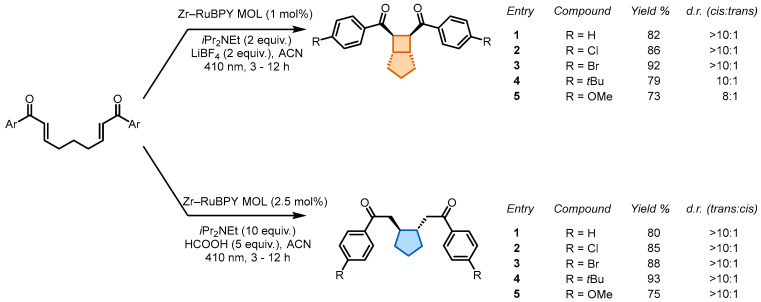
The [2+2] photocycloaddition and reductive coupling of aryl bisenones, promoted by a metal–organic layer (MOL), Ru(II)-based photocatalyst.

In the field of heterogeneous catalysis, Lin and co-workers introduced a metal–organic layer (MOL)-based photocatalytic platform that overcomes diffusion and pore-size limitations that are common in 3D metal–organic frameworks (MOFs) [25]. The authors report the synthesis of a Zr–RuBPY MOL that incorporates [Ru(bpy)_3_]^2+^ photosensitizers into a two-dimensional, crystalline structure with a monolayer thickness of ~1–2 nm. This hybrid catalyst efficiently promotes both intramolecular and intermolecular [2+2] cycloadditions of bisenones (Scheme 14) under visible-light irradiation, giving the corresponding cyclobutene adduct in 73–92% yields with high diastereoselectivity (up to >10:1 *cis*:*trans*). Furthermore, the Zr–RuBPY MOL retained its structure and catalytic activity across multiple runs, with minimal Ru leaching (<3%), confirming its robustness.

**Scheme 15:**
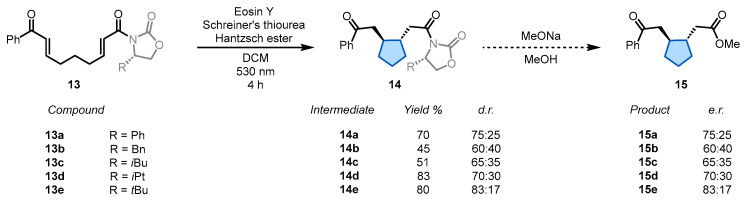
Diastereoselective reductive coupling of aryl bisenones.

In 2021, Benaglia et al. [26] hypothesized that it could be possible to direct the radical attack employing a chiral oxazolidinone as a chiral auxiliary, to develop a novel stereoselective cyclization of (bis)enones for the synthesis of the *trans* 5-*exo*-trig product. Inspired by the work of Zeitler, the authors optimized the reaction conditions for the new substrates, using Eosin Y as a photocatalyst, Schreiner’s thiourea as an H-bond donor to activate the aryl enone and the Hantzsch ester as a sacrificial reducing agent in dichloromethane as a solvent. The reaction was also tested with different chiral oxazolidinones, with the purpose of maximizing the stereocontrol. The reaction mixture was irradiated for 4 h with green LED (530 nm) at room temperature. The reaction achieved good to excellent yields (up to 83%); in the meantime, the presence of a fixed stereocenter favored one diastereoisomer over the other, obtaining an 83:17 *d.r.* when the tert-butyl oxazolidinone was used (Scheme 15, **14e**). Separation of the two diastereoisomers by column chromatography and subsequent removal of the chiral auxiliary allowed to recover the cyclopentane adduct **15e** in 83:17 *e.r*.

HPLC analysis on the chiral stationary phase confirmed that the enantiomeric ratio of the products after auxiliary removal (Scheme 15, **15a**–**e**) was identical to the diastereoisomeric ratio evaluated by NMR on purified products **14a**–**e**. The authors also were able to transpose the reaction under flow conditions, improving both the yield and productivity, achieving 87% yield in 40 min of residence time without affecting either the diastereoselectivity or the enantioselectivity.

In 2023, Goddard et al. studied the possibility of using the supported Eosin Y photocatalyst for promoting the [2+2] photoredox cyclization of aryl bisenones under visible light [27]. The study focuses on how different mesoporous silica architectures affect the course and outcome of the photochemical reaction.

**Scheme 16:**
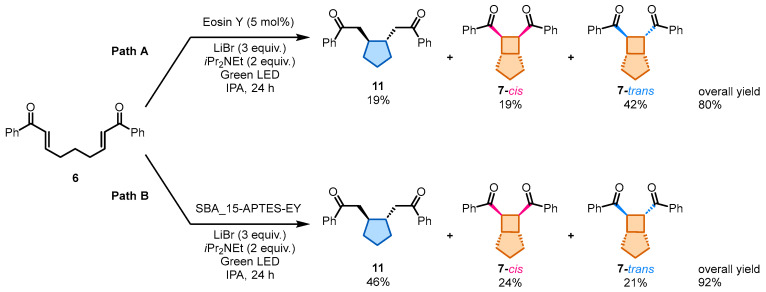
Influence of selectivity for the reductive coupling of compound **6**, using SBA-15-APTES-EY as the heterogeneous catalyst.

When carried out with the immobilized Eosin Y, the reaction showed that the nature of the silica support has a decisive impact on the selectivity between the two product types (mono- or bicyclic). The authors compared the results achieved using the free photocatalyst Eosin Y (Scheme 16, Path **A**) and the SBA-15-APTES-EY (Scheme 16, Path **B**) catalyst (characterized by its cage-like mesoporous framework). It was observed that the mesoporous catalyst was able to improve the formation of the 5-*exo*-trig adduct **11**, which was the minor product in the aforementioned methodologies (Scheme 16). The study demonstrates that in heterogeneous photoredox catalysis, the support material plays a key role in the radical process.

**Scheme 17:**
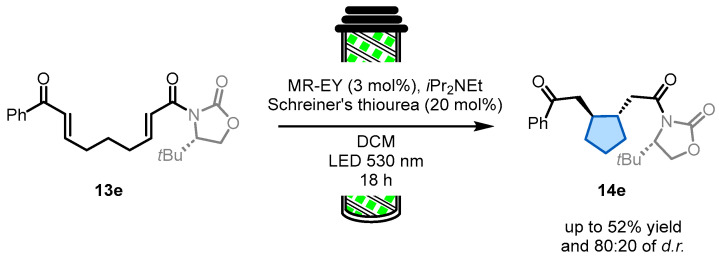
Diastereoselective reductive coupling of aryl bisenones with supported Eosin Y photocatalyst (MR-EY).

The use of supported Eosin Y was further studied one year later by Rossi and co-workers for the synthesis of enantiomerically enriched cyclopentane rings through the use of chiral auxiliaries [28]. The authors report the possibility of supporting Eosin Y on Merrifield resin (MR-EY) to enhance the recycling of the catalyst (Scheme 17). The authors achieved the desired cyclization for both the symmetric and non-symmetric aryl enones after 18 h of irradiation, using 3 mol% of MR-EY in good yields. The supported catalyst can be used up to three times, before being regenerated by irradiation by green light for 1 h, blowing in air at 1 atm.

**Scheme 18:**
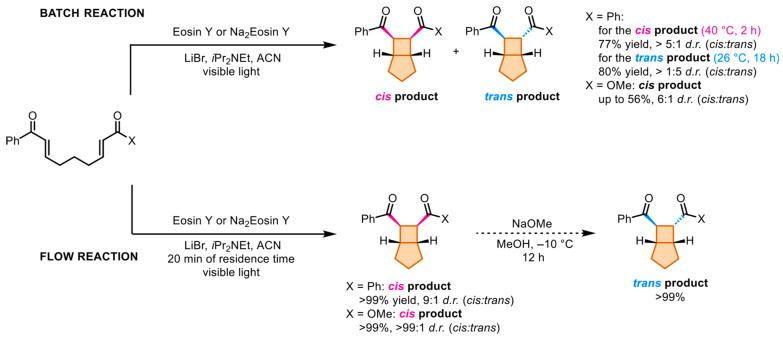
The [2+2] photocycloaddition of aryl enones under batch and flow conditions.

Taking inspiration from the works of Yoon and Zeitler [18,21] in 2024, Rossi and co-workers focused their attention on the synthesis of bicyclo[3.2.0]heptanes by using Eosin Y as a photocatalyst, exploring both batch and continuous-flow conditions [29]. The authors first optimized the reaction under batch conditions, using green LEDs and a compact fluorescent lamp (CFL) as light sources. Temperature and reaction time play a crucial role in determining product selectivity; at 40 °C for 12 h, the *trans* isomer predominates, while at a lower temperature (26 °C) and for a shorter reaction time (2 h), the *cis isomer* becomes the major product (Scheme 18).

To simplify the catalyst recovery, Eosin Y was also immobilized on a Merrifield resin, providing a heterogeneous catalytic system that maintained good reactivity, though with slightly reduced yields due to light penetration limits. Moreover, the researchers successfully transferred the methodology with the free photocatalyst to continuous-flow conditions, combining photoredox catalysis with flow technology to enhance efficiency and scalability. Under flow conditions, quantitative conversion and excellent *cis* selectivity (up to >99:1 *cis*:*trans*) were observed. The continuous-flow approach significantly increased the space–time yield, which was up to 50 times higher than in batch reaction, and enabled easy scale-up to gram-scale while maintaining high yields (up to 97%) and stereocontrol.

**Scheme 19:**
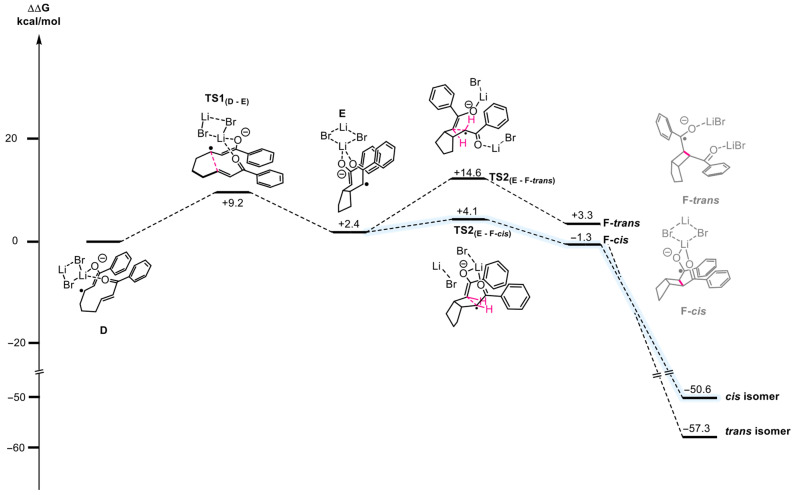
Gibbs free energy profiles (in kcal mol^−1^) for the anion radical [2+2] photocyclizations starting from radical anion A, computed at the M062X/6-31G(d,p)/PCM (ACN) level. The *cis* pathway is highlighted in light blue.

To rationalize the observed selectivity, DFT calculations were performed, confirming that the SET pathway is favored over energy transfer, and that LiBr plays a key role in lowering the reduction potential of the enone. The calculations also revealed that the formation of the *cis* product is the more favorable pathway (highlighted in blue, Scheme 19) for the [2+2] cycloaddition; on the other hand, the *trans* isomer is thermodynamically more stable (Scheme 19). From the DFT calculations, the authors suggest that the [2+2] cycloaddition proceeds preferentially through the formation of the *cis* isomer, which explains why it is obtained as the major product at shorter reaction times. The authors also investigated the possible mechanism leading to the formation of the *trans* product at longer reaction times. This was attributed to an isomerization of the *cis* isomer into the *trans* one, which was promoted by a side product generated during the reaction.

**Scheme 20:**
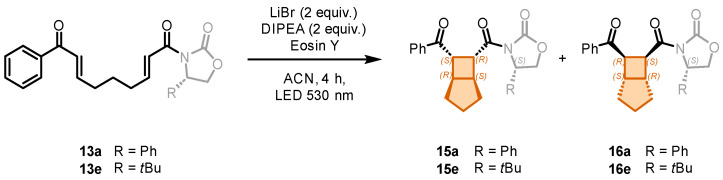
Diastereoselective [2+2] photocycloaddition of aryl enones.

One year later, Rossi and coworkers published a paper on the enantioselective version for the organophotoredox-catalyzed synthesis of bicyclo[3.2.0]heptanes through the anion radical [2+2] photocycloaddition of aryl bisenones [30]. As previously reported for the monocycle product, the use of chiral oxazolidinone auxiliaries attached to the enone substrates enables enantioenriched products, achieving both high chemical yields and stereocontrol. In the study, bisenones **13a** and **13e** (Scheme 20), each bearing different oxazolidinone units, were synthesized via a sequence involving Wittig reactions, acylation and condensation steps. Under green LED irradiation (530 nm) and in the presence of Eosin Y (0.5–1 mol%) and LiBr, compounds **13a** and **13e** underwent efficient cyclization to form bicyclo[3.2.0]heptanes **15** and **16**. The authors report that the [2+2] cyclization reaction of non-symmetric bisenones could undergo four different closure-type radical reactions—two for the formation of the first cycle: *syn* and *anti* attack, and two for the formation of the cyclobutene ring: *cis* and *anti* attack—leading to the formation of four classes of products: *cis*-*anti*, *trans*-*anti*, *cis*-*syn* and *trans*-*syn* derivatives, respectively. Due to the presence of a defined stereocenter on the chiral auxiliary, 16 different stereoisomers can be formed during the reaction.

Notably, the reactions gave moderate-to-high yields (up to 76%) and good diastereoselectivity (up to 78:22 *d.r.*), with only the two *cis*-*anti* isomers (Scheme 20, products **15** and **16**) as the sole products. The removal of the chiral auxiliary resulted in the formation of the two corresponding enantiomers. Structural analysis by single-crystal X-ray diffraction confirmed the absolute stereochemistry of the major product **16a** as (**1S**, **5R**, **6R**, **7S**). NMR analysis further supported that the reaction selectively yields *cis*-*anti* bicycloadducts, distinguishing it from previously known systems that also form *trans*-*anti* isomers.

To elucidate the mechanism and stereochemical outcome, the authors performed DFT calculations of all the possible products, which showed excellent agreement with the experimental data.

**Scheme 21:**
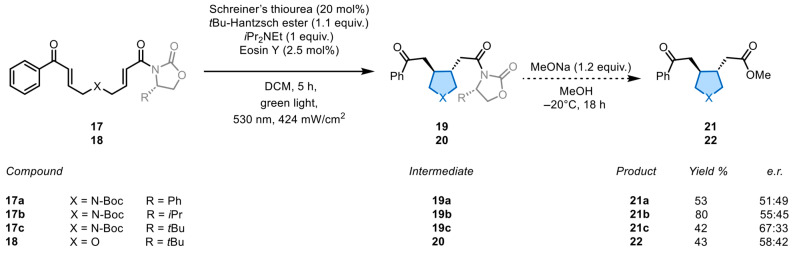
Diastereoselective reductive cyclization of nitrogen- and oxygen-containing aryl enones.

More recently, Benaglia et al. reported the possibility to expand the use of chiral oxazolidinones as auxiliary in the radical-promoted cyclization of bisenones also for the stereoselective synthesis of heterocyclic targets: in particular, for the intramolecular reductive cyclization of nitrogen and oxygen-containing analogs aryl-enones [31]. The reaction was carried out using Eosin Y as the photocatalyst, Schreiner’s thiourea as a hydrogen bond donor and irradiation at 530 nm; moreover, a mixture of Hantzsch ester and *i*Pr_2_Net sacrificial reducing agents was necessary. The reaction was tested with different chiral oxazolidinones. The best stereoselectivity was achieved with the *t*Bu-substituted one, yielding enantiomeric ratios (after removal of the chiral auxiliary) of 67:33 *e.r.* for compound **21c** and 58:42 *e.r.* for compound **22**. All results are reported in Scheme 21.

In general, photocatalytic cyclization reactions of (bis)enones represent the most extensively studied processes. These systems exploit the enhanced redox reactivity of photocatalysts in their excited states, thereby enabling transformations that would otherwise be inaccessible. Both metal-based photocatalysts and organic dyes have been successfully employed, often affording excellent results. As discussed in the reported examples, intramolecular cyclization of (bis)enones can lead to two distinct products. Notably, the reaction outcome can be controlled through modulation of the reaction conditions: five-membered ring products are typically formed when a neutral radical is involved in the mechanism, whereas bicyclic products arise when a radical anion serves as the key intermediate [19,25]. Despite these advantages, complete regioselectivity during ring closure is not always achieved, and considerable effort is still required to address this limitation.

The use of light to promote chemical reactions suffers from an inherent limitation associated with light penetration, which complicates the scale-up of photochemical processes [32]. To address this challenge, particularly in the cyclization of (bis)enones, the integration of photocatalysis with flow chemistry has emerged as a promising strategy, opening new avenues for reaction development [33]. This combination has been investigated using multiple approaches, including both supported and homogeneous photocatalytic systems [26,29]. More recently, an enantioselective variant of the reaction has been explored through the use of chiral auxiliaries to provide stereochemical control [26,28,30,31]. However, only modest levels of enantioselectivity have been achieved to date, leaving significant room for further improvement in this area.

Overall, these studies highlight how photoredox catalysis has evolved into a versatile and finely tunable platform for controlling the reactivity, regioselectivity and stereochemical outcome of (bis)enones cyclization. The combination of mechanistic insight, catalyst design and computational studies has enabled the development of increasingly selective, sustainable and scalable methodologies for the construction of complex cyclic architectures.

## 3. Metal-Catalyzed Methodologies

While photochemical strategies enable redox activation under mild conditions through light-induced excited states, alternative approaches rely on the intrinsic reactivity of metal centers. In this context, metal-driven processes offer complementary pathways for substrate activation, exploiting metal–substrate interactions and redox cycles to achieve transformations under a distinct, yet complementary, mechanistic framework. Since the dawn of modern chemistry, metals have played a crucial role in the development of synthetic organic chemistry. Among the most significant examples are metal-catalyzed cross-coupling reactions [34]. More recently, metals also played a crucial role in the development of radical chemistry, both for generating radicals and for improving the selectivity of reactions involving radical intermediates. To a limited extent, they also played a role in advancing the application of bisenone reactivity via single-electron transfer (SET) processes.

**Scheme 22:**
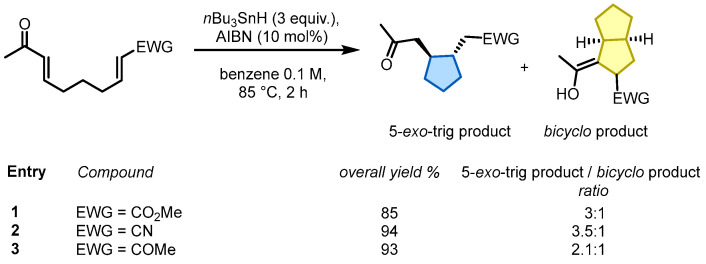
Cyclization of enones, promoted by *n*Bu_3_SnH.

To the best of our knowledge, the first example was reported by Enholm and co-workers in 1995, in which *n*Bu_3_SnH induced the intramolecular cyclization of enones, leading to the formation of a mixture of cyclized products (Scheme 22) [35].

**Scheme 23:**
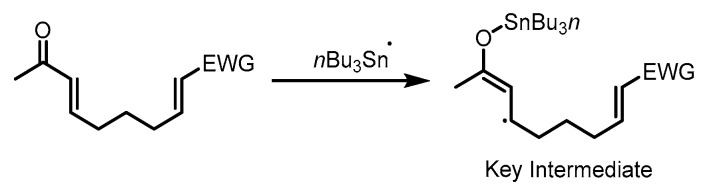
Representation of the key intermediate.

The organometallic species was used in large excess (three equivalents), which was relative to the starting bisenones; moreover, the presence of a radical initiator, such as azobisisobutyronitrile (AIBN), was required. A modest scope was achieved, with overall yields of up to 80%. It was noticed that decreasing the reagent concentration favors the monocyclic product. For example, in the reaction depicted in Scheme 22, simply diluting the solution from a 1 M solution to 0.01 M increases the ratio from 9:1 to over 50:1. As for the mechanism, the authors postulate the presence of a tin enolate as a key intermediate, which was formed after SET to the reagent and consequent radical formation (Scheme 23).

**Scheme 24:**
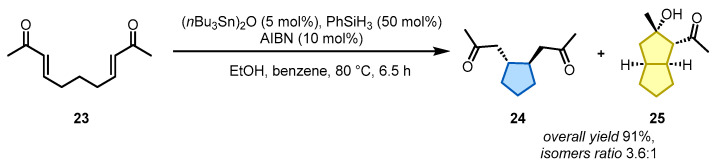
Metal-catalyzed cyclization of bisenones.

In a concentrated solution, the higher availability of tin hydride allows the monocyclic tin enolate to undergo a second intramolecular cyclization by the attack from the carbon in α position to the ketone on the electron-withdrawing group. A year later, Fu et al. demonstrated that the intramolecular cyclization of bisenones could be achieved by using the metal species in a catalytic amount [36]. This was possible due to the use of an additive, PhSiH_3_, that could regenerate the organotin catalyst (Scheme 24).

Organotin oxide was used as a convenient and cost-effective source of tin hydride, generated through its reaction with EtOH. Mechanistically, the process follows the pathway described by Enholm and co-workers [35], with the additional step of regenerating the tin catalyst by hydride transfer from the silane, forming the corresponding silylenolether. In a similar application, Parsons’s group reported the synthesis of substituted tetrahydrofurans from the corresponding (bis)enones ether, using a stoichiometric amount of tributyltin hydride [37].

**Scheme 25:**
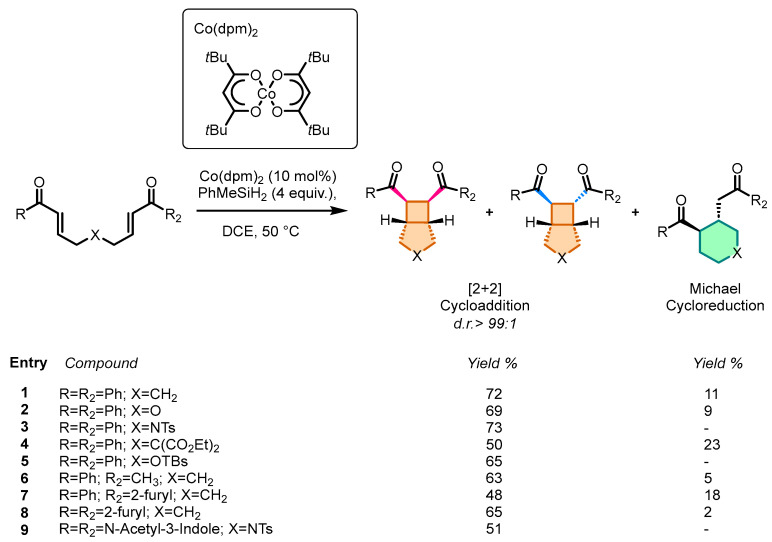
Cobalt(II) catalyzed [2+2] cycloaddition.

In 2001, Krische and co-workers demonstrated that Co(II) complexes could also serve as the key catalyst for the cyclization of aryl bisenones [38]. This strategy, presented in Scheme 25, was highly efficient in terms of yield and diastereoselectivity, leading to the formation of the [2+2] cycloaddition product in good yields and outstanding diastereoselectivity (*d.r.* > 99:1).

**Scheme 26:**
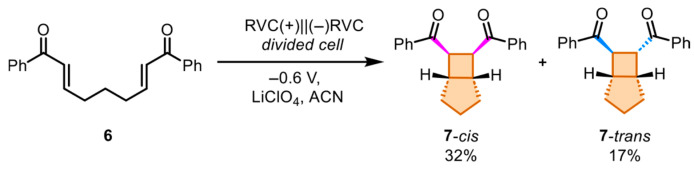
Electrochemical [2+2] cycloaddition.

By expanding the reaction scope, the authors discovered that the reaction tolerated different aromatic and heteroaromatic ketones, as well as the presence of ramification on the linear chain, in yields between 50% and 73%. Notably, in all the reported examples, only the *cis* isomer was obtained. In addition, other than the [2+2]-cyclized product, in some cases, the formation of the Michael addition by-product was observed. The authors integrate their results with different control experiments, revealing, in particular, that even when starting from a mixture of E and Z bisenone, only the *cis* isomer was obtained, suggesting the formation of a radical as an intermediate in both cases. Nevertheless, treatment of the *cis* product with a Brønsted or Lewis acid (such as TFA or BF_3_) led to its conversion into the *trans* isomer. According to the proposed mechanism, the excess of silane is necessary to pre-activate the catalyst, as it coordinates with the double bonds, thereby restricting the configurational flexibility of the transition-state geometry, which directs the cycloaddition to produce a single isomer. To demonstrate the role of the metal in stereoselectivity, the same reaction was repeated under electrochemical conditions in the absence of the Co(II) complex (Scheme 26), confirming the radical nature of the reaction and yielding a mixture of isomers.

**Scheme 27:**
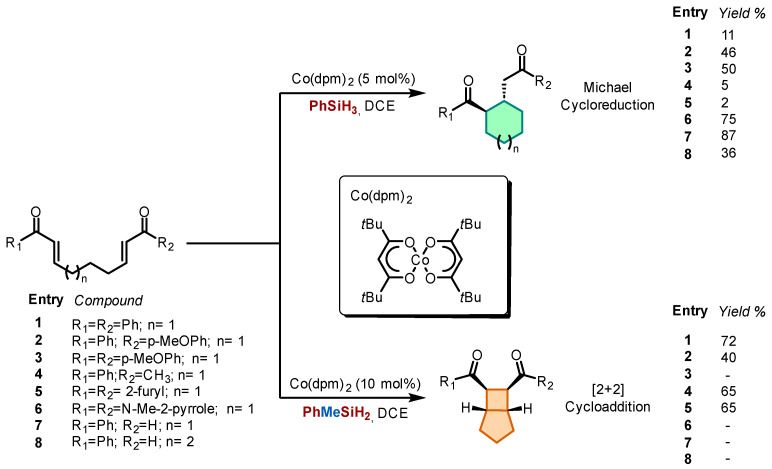
Possible reaction pathways.

In the following year, Kirsche’s group published a paper focused on the mechanistic investigation of the diastereoselective cycloreduction and cycloaddition of mono- and bisenones promoted by the Co(dpm)_2_ complex in the presence of differently substituted silanes (Scheme 27) [39].

**Scheme 28:**
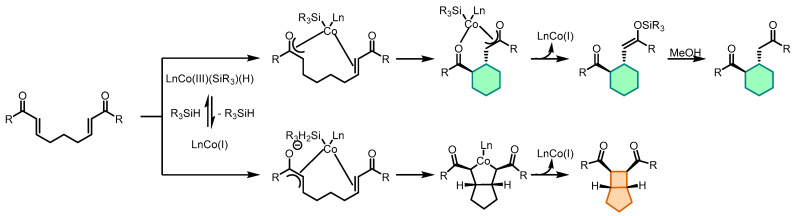
Proposed mechanism pathways.

By combining data from deuterium labeling experiments, electrochemical tests, crystallographic characterization of some intermediates and blank tests, the authors proposed the following steps for the two mechanisms, demonstrating that the choice of silane is a critical factor. When phenyl silane was used, the oxidative addition favored the hydrometallative pathway, promoting cycloreduction. In contrast, using a bulkier silane, such as PhMeSiH_2_, the oxidative addition was disfavored, thereby stabilizing the Co(I) center and leading to [2+2] cycloaddition via radical formation (Scheme 28).

**Scheme 29:**
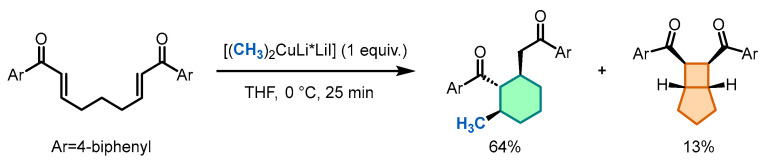
Model reaction for the Gilman reagent investigation.

In 2004, Kirsche’s group used the aryl bisenones as a mechanistic probe in the cyclization reactions catalyzed by the Gilman reagent, Me_2_CuLi*LiI [40]. In the early years of the 21st century, Gilman’s reagent was at the center of debate over its mechanism of action: in particular, whether it proceeded through an intermediate containing a dystonic radical, thereby involving a possible electron transfer. Following the results reported in the aforementioned paper, Kirsche and co-workers observed that reduced bisenones, which are prone to [2+2] cyclization via chemical or cathodic electron transfer, are ideal probes for detecting an anionic radical. The reaction was conducted in THF at 0 °C with one equivalent of the Gilman reagent. Two products were obtained, one from the [2+2] cycloaddition and the other from methylation, as depicted in Scheme 29.

The test, as expected, confirmed the presence of the electron-transfer product; however, the major product was the methylated derivative. Interestingly, changing the amount of organocuprate led to a significant shift in the product ratio. Indeed, when a large excess (200 mol%) was used, only the methylated derivative was obtained in the 85% yield; on the contrary, when a catalytic amount (25 mol%) was used, the ratio was inverted, with only the [2+2] cyclization product obtained in 91% yield. Moreover, when a stoichiometric amount was used at a lower concentration, the electron-transfer product was the major product. Based on these results and additional data from further experiments, they concluded that the form in which the Gilman reagent is present in solution dictates the reaction outcome: at low concentration or mol% loading, the monomeric form predominates, favoring the electron transfer to form the distonic radical on the bisenones and consequent formation of the bicyclic product; at higher concentration or higher mol% loading, the dimeric dilithium aggregate is formed, favoring the formation of the methylated derivatives.

**Scheme 30:**
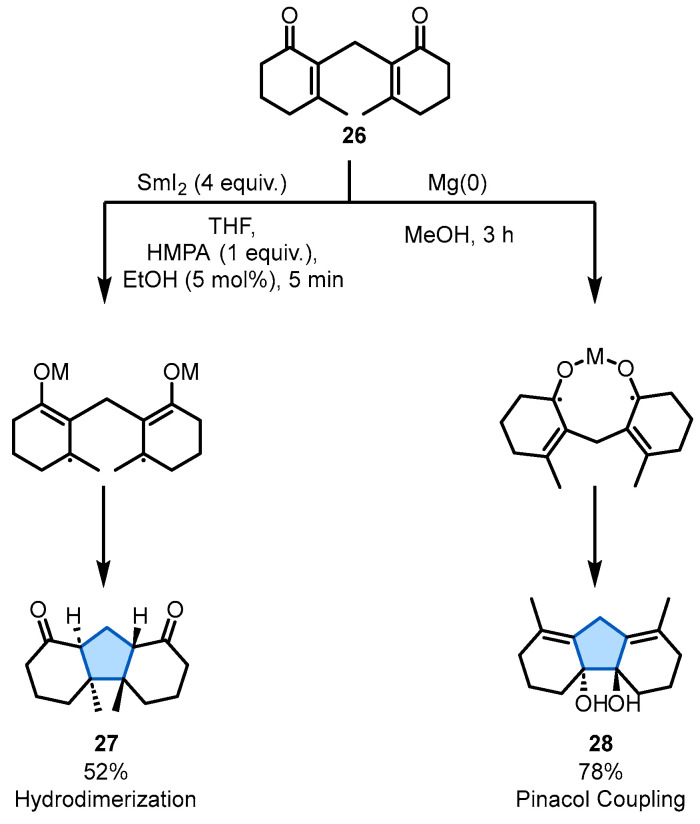
Mechanistic rationale.

In 2005, Omune and co-workers published a study investigating the intramolecular hydrodimerization and pinacol coupling of cyclic bisenones promoted by metals complexes [41]. Two different cyclized products were obtained depending on the metal used: with SmI_2_, the hydrodimerisation derivative is formed, while Mg(0) favored pinacol coupling (Scheme 30). This selectivity arises from the formation of the different metal-chelated intermediate: two molecules of samarium diiodide were necessary to reduce both carbonyl groups, so the chelation played a marginal role in the mechanism, whereas a single magnesium atom could reduce both the carbonyl groups, resulting in a chelation process. Based on these results, Omune and co-workers subsequently published a study that further examined the methodology and expanded its scope [42].

**Scheme 31:**
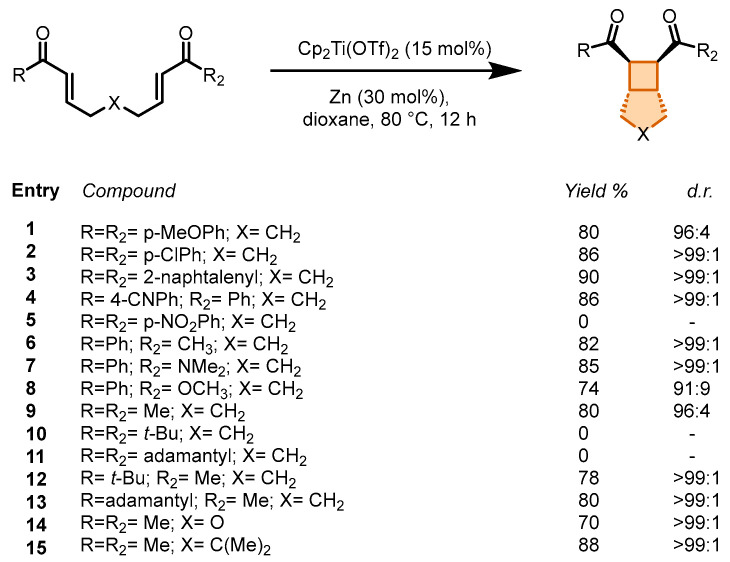
Titanocene catalyzed [2+2] cyclization.

More recently, Gansauer’s group used titanocene to promote the [2+2] cyclization of bisenones (Scheme 31) [43].

The Ti(IV) precatalyst was reduced in situ by zinc to form the active species Ti(III), which, upon coordination with the bisenone, performed the electron transfer needed to generate the radical. After cyclization, back-transfer from the ketyl radical to the titanocene dissociation took place, regenerating the Ti(III) catalyst. A comprehensive study on bisenones with various substituents, including aliphatic, aromatic and mixed residues, was performed, leading to the corresponding products with the *syn* configuration.

In summary, these examples describe a range of intramolecular cyclization reactions employing different metals, including Sn, Co, Sm, Mg and Ti. In contrast to photocatalytic methods, the diversity of the accessible products is markedly broader and strongly dependent on the specific metal employed. Notably, the formation of six-membered ring products following the pathway depicted in Figure 2 is observed exclusively in metal-mediated reactions. Moreover, as in photocatalytic processes, selective formation of a given cyclization product can be achieved through careful modulation of the reaction conditions (Scheme 27). With respect to diastereoselectivity, metal-catalyzed methodologies in [2+2] cycloadditions exhibit exceptional control (*d.r*. up to >99:1), reaching diastereomeric ratios that are generally unattainable under photochemical conditions. The development of enantioselective variants of these metal-catalyzed processes, potentially through the use of chiral metal complexes, represents a promising and largely unexplored area.

## 4. Electrochemical Methodologies

While metal-driven processes rely on the redox activity of metal centers to mediate substrate activation, electrochemical methods achieve analogous transformations by directly controlling electron transfer at the electrode surface. This shift from molecular to electrode-based redox control offers new opportunities for tuning reactivity and selectivity. Over the past decade, electrochemistry has experienced a renewed interest as a valuable tool in synthetic chemistry. Electrochemistry offers a very mild and atom-efficient method to achieve selective oxidative or reductive transformations, using electrons as traceless reactants, and thus removing the needs for harsh conditions, and the use of typically toxic reducing and oxidizing agents [44]. The ability to carry out redox processes without aggressive reagents has led to a significant research activity in this area, which resulted in an increased number of publications in the field of electrochemistry, kicking off the renaissance of this topic [45]. Notably, electrochemical methods enable the formation of C–C, C–O, C–N, C–heteroatom and heteroatom–heteroatom bonds in an efficient and sustainable way [46].

**Scheme 32:**
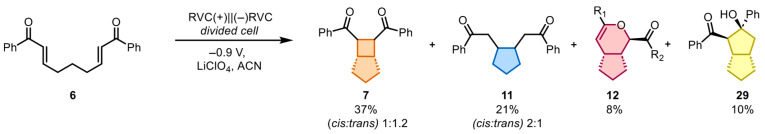
Cathodic reduction of compound **6**.

In this context, the possibility of generating the anion radical of several bisenones under electrochemical conditions was investigated. The first example is dated in 2002, when Krische and Bauld, in parallel with their study on an intramolecular cobalt-catalyzed [2+2] cycloaddition of tethered enones (see Section 2), reported the cathodic reduction of different bisenones [47], with the primary goal of demonstrating the formation of the anion radical. Cyclic voltammetry performed on the model bisenone **6** revealed that the peak reduction potential of **1a** was −1.20 V vs. SCE and irreversible. A 0.02 M solution of **6** in electrolyte solution (0.1 M LiClO_4_ in ACN) at room temperature was subjected to electrochemical reduction. The overall yield of the isolated products was 76%, with the remaining mass accounted for by polymeric by-products. Analysis of the isolated products mixture revealed that the main products were the bicyclic isomers **7-*cis*** and **7-*trans***, as well as the Diels–Alder cycloaddition derivative **12** with a 2:2.4: **7-*cis*:7-*trans*:12** ratio. In addition, monocyclic product **11** and the corresponding aldol product **29** were formed in 21% and 10% yields, respectively. The results of this initial study are reported in Scheme 32.

**Scheme 33:**
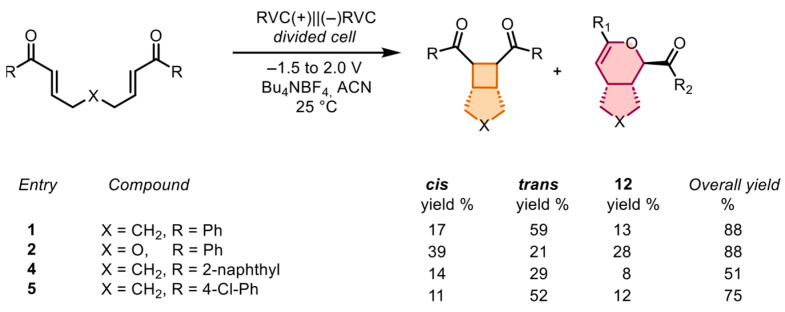
Electrocatalytic cyclobutanation reactions of various bis(enones).

The attempts to expand the scope of cathodic reduction to other bisenones failed, except for the asymmetric substrate bearing Ph and Me as substituents, which afforded the desired cycloaddition products in 35% yield as *cis* and *trans* mixture. This study provided new examples of a relatively uncommon reaction type, anion radical chain cyclobutanation, and a starting point for broadening the scope of these new reaction types. Some years later, the same authors reported the intramolecular cyclobutanation of bisenones via homogeneous electron transfer [48]. The reaction was chemically induced by the addition of an anion radical (chrysene) to bisenones, but for selected substrates, electrochemical promotion of the cycloaddition was examined. Both *cis*- and *trans*-cyclobutane isomers were formed, in good total yields of pericyclic products. However, the electrochemical reaction also led to the formation of pericyclic product **12** in 10 to 30% yield (Scheme 33).

**Scheme 34:**
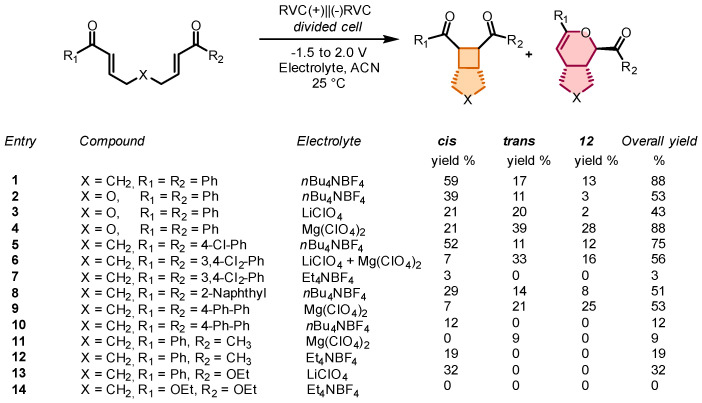
Screening of conditions for the electrochemically induced cyclization of bisenones.

In the same year, Bauld and co-workers published a study entirely focused on the electrochemical study of intramolecular anion–radical cyclobutanation reactions [49]. In this paper, the authors reported the development of more efficient conditions than those previously presented [48]. The use of tetraalkylammonium tetrafluoroborate electrolytes in acetonitrile proved to be essential for delivering product **7** in a high yield, with a *cis*:*trans* ratio of up to 3.5:1. Nevertheless, a fine-tuning of the reaction conditions was necessary for each substrate bearing an electron-donating or electron-withdrawing substituent to maximize the outcome. Furthermore, it was demonstrated that at least one aryl group as a substituent on the bisenone is required for the reaction, with optimal results obtained when both positions are substituted with aryl groups (Scheme 34). The authors attributed this observation to the greater delocalization and stabilization of the radical anion formed upon reduction of the α,β-unsaturated ketone bearing an aryl substituent. This enhanced stabilization likely resulted in a lower reduction potential for these substrates, relative to analogs bearing two methyl or two ester substituents.

**Scheme 35:**
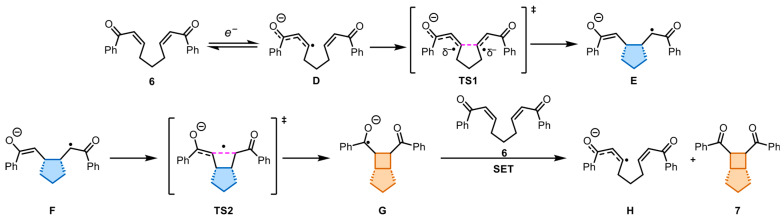
Proposed mechanism for the anion radical chain cyclobutanation of compound **6**.

Evidence for the formation of a distonic anion radical intermediate was provided by trapping it through protonation, which was achieved by adding a slight excess of acetic acid at the beginning of the electrolysis. On these bases, a reaction mechanism for this transformation was proposed, which is illustrated in Scheme 35.

According to this mechanism, the reduction of the bisenone at the cathode leads to anion radical, **D**, which then cyclizes to the distonic anion radical intermediate **E** (Scheme 35). This intermediate cyclizes to the anion radical of the cyclobutane product, **F**. The anion radical moiety presumably is localized upon one of the benzoyl groups. Finally, exergonic single-electron transfer (SET) from **F** to bisenone **6** sets up the chain process affording the neutral cyclobutane product **7**. The distonic anion radical intermediate **E**, can also cyclize to a Diels–Alder adduct anion radical, where the anion radical moiety again resides upon a benzoyl moiety.

The same authors demonstrated that the nature of the supporting electrolyte cation plays a crucial role in controlling the diastereoselectivity of electro-reductive intramolecular cycloaddition reactions of bisenones [49]. Systematic variation in perchlorate electrolytes containing mono- and divalent cations (Li^+^, Na^+^, K^+^, Mg^2+^ and Ba^2+^) revealed a strong correlation between cation chelation ability and product distribution. Divalent cations strongly favor the formation of *cis*-cyclobutane products by stabilizing the distonic anion radical intermediate through bidentate coordination to the carbonyl oxygen atoms while suppressing the thermodynamically favored *trans* isomer. Remarkably, Ba^2+^ completely inhibits *trans*-cyclobutane formation and simultaneously enhances the competing Diels–Alder cycloaddition pathway: an effect attributed to optimal size-matching between the cation and the chelation cavity of the intermediate. These findings highlight the effects of electrolyte cations as a powerful parameter for steering selectivity in electrochemical pericyclic reactions.

**Scheme 36:**
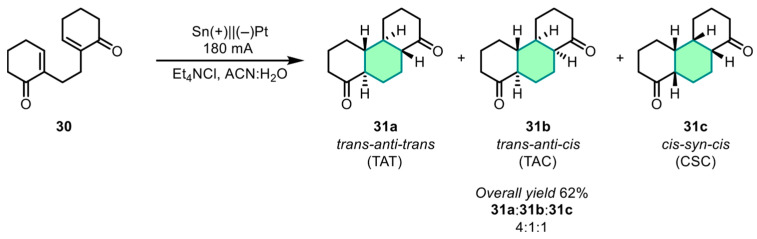
Electrochemical reductive cyclization of compound **30**.

In 2007, Omune and Handy reported the possibility to exploit the reductive coupling cyclization of α,β-unsaturated enones for the synthesis of tricycle **31** (Scheme 36) [42]. The reaction was carried out under electrochemical conditions, using a tin cathode and a platinum anode, with tetraethylammonium chloride as a supporting electrolyte in a mixture of water and acetonitrile as a solvent system (Scheme 36).

The authors report the formation of a mixture of three isomers of product **31**, with the major one being *trans*-*anti*-*trans* (TAT, **31a**). The reaction scope was also expanded to other substrates, achieving high yields of up to 73%. The reaction was also investigated under different conditions: metal-mediated and photochemical, as discussed in the previous chapters.

The primary objective of electrochemically-driven cyclization reactions was to demonstrate the formation of radical anion intermediates that were analogous to those generated under photocatalytic or metal-mediated conditions. At the moment, this approach has been largely limited to (bis)enone substrates bearing at least one aryl substituent, which is likely due to the lower reduction potential required for their activation. Reaction outcomes are generally more difficult to control, and, in all the reported cases, mixtures of products are obtained.

In conclusion, electrochemical methodologies currently represent the least developed approach among those discussed; however, they offer significant potential for future investigation and methodological advancement.

## 5. Current Challenges and Limitations

Despite significant advances in methods for radical generation, several challenges persist across the *photochemical*, *metal-mediated* and *electrochemical* approaches. Limitations in substrate scope remain a primary hurdle, as the presence of at least one aryl substituent on the (bis)enone appears to be necessary to stabilize the generated radical and enable further reaction. In addition, sensitive functional groups are often prone to over-reduction or decomposition under photochemical or redox-active conditions, further restricting the applicability of these cyclization reactions.

Cost considerations further limit the widespread adoption of photochemical and electrochemical methodologies when compared with more traditional metal-mediated approaches. Photoredox catalysis often relies on expensive ruthenium- or iridium-based complexes, which increases costs and raises concerns regarding scalability. Similarly, electrochemical methods typically require specialized instrumentation and carefully controlled setups, adding operational complexity. In contrast, many metal-mediated processes employ relatively inexpensive and readily available metals and can often be implemented by using simpler experimental setups, making them more practical and accessible. However, enantioselective variants of these metal-catalyzed processes are still underdeveloped.

The attempts at developing supported catalysts for addressing catalyst recyclability revealed that the nature of the support plays a key role in determining the reaction outcome. Moreover, photocatalytic systems often suffer from photobleaching, leading to catalyst deactivation.

Scalability remains a significant challenge across photochemical, electrochemical and metal-mediated methodologies. In photochemical reactions, poor light penetration in larger volumes limits reaction efficiency, whereas in electrochemical systems, electrode fouling and mass-transfer limitations can compromise reproducibility and long-term operation. Metal-mediated processes, while generally being more robust, are not immune to scale-up issues, considering catalyst cost and reaction exothermicity. The integration of flow chemistry could provide a versatile solution, enabling uniform light exposure, improved mass and heat transfer and continuous operation. Flow-based approaches not only enhance scalability and reaction control for photochemical and electrochemical systems but can also improve safety, efficiency and reproducibility in metal-mediated reactions, offering a general strategy toward viable implementations of the discussed transformations.

## 6. Summary and Outlook

α,β-Unsaturated (bis)enones have emerged as highly versatile building blocks for the construction of cyclic frameworks via intramolecular single-electron transfer (SET) processes. Their distinctive electronic structure—combining a conjugated π-system with an electron-deficient carbonyl moiety—facilitates efficient radical generation under photochemical, electrochemical and metal-promoted activation. Over the past decade, these substrates have enabled a diverse array of cyclization reactions, broadening the access to carbo- and heterocycles of different ring sizes with excellent functional-group tolerance and high atom economy. Mechanistic investigations have clarified the crucial role of enone (and bisenone) geometry, substituent effects and redox properties in steering the selectivity and efficiency of SET-induced cyclizations. As a result, α,β-unsaturated (bis)enones now occupy a central position in modern radical-based synthetic strategies aimed at rapid molecular complexity generation.

Moreover, great interest has been devoted to controlling the selectivity and stereochemical outcome of the radical-promoted cyclization of α,β-unsaturated ketones. In particular, the use of specific solvents and additives has proven to be fundamental for controlling the cyclization pathway. Protic solvents and Brønsted acid activation favor the formation of the 5-*exo*-trig adduct (compound **VI**, Figure 2), which is obtained exclusively in the *trans* configuration. Conversely, aprotic solvents and Lewis acid activation lead to the formation of the [2+2] bicyclic product (compound **X**, Figure 2) as a mixture of diastereoisomers (*cis*/*trans*). Researchers have demonstrated that it is possible to control the diastereoselectivity of the [2+2] cyclization pathway by adjusting the temperature and reaction time.

Additionally, alternative cyclization pathways can be promoted by coordinating the carbonyl moiety present in the substrate with bivalent metals (compound **IV**, Figure 2). For example, the formation of the Diels–Alder bicyclic product (compound **XII**, Figure 2) is preferentially achieved under electrochemical conditions using Mg(ClO_4_)_2_ as the electrolyte (see Scheme 34). The possibility of controlling stereoselectivity has also been investigated through the use of chiral auxiliaries and DFT calculations, leading to interesting results and important information about the radical intermediate stability, spin densities and cyclization pathways.

However, despite significant progress, the full potential of α,β-unsaturated (bis)enones in intramolecular SET-mediated cyclization remains far from exhausted. Expanding the methodology toward more robust enantioselective variants still represents a particularly attractive challenge. The integration of SET-driven cyclizations into multistep cascade processes could substantially enhance synthetic efficiency. Furthermore, the intramolecular SET-mediated cyclization of (bis)enones would greatly benefit from the use of continuous-flow methodologies in terms of safety, scalability and control of reaction parameters. Advances in computational tools and ultrafast spectroscopy will also deepen mechanistic understanding, enabling the rational tuning of redox potentials and conformational preferences in (bis)enone substrates. Overall, α,β-unsaturated (bis)enones are poised to continue shaping innovative radical methodologies, providing increasingly powerful tools for the streamlined synthesis of complex cyclic molecules.

## Data Availability

No new data were created or analyzed in this study. Data sharing is not applicable to this article.

## List of Abbreviations

The following abbreviations for solvents are used in this manuscript:

DMF	*N*,*N*-dimethyl-formamide
IPA	2-Propanol
ACN	Acetonitrile
DCM	Dichloromethane
EtOH	Ethanol
DCE	1,2-Dichloroethane
THF	Tetrahydrofuran
